# A Rare Case of Niemann-Pick Disease Type-A

**DOI:** 10.7759/cureus.59427

**Published:** 2024-04-30

**Authors:** Faiza Gul, Sapna Begum, Palwasha Rasool, Safdar Shah, Muhammad Waqar

**Affiliations:** 1 Paediatrics, Lady Reading Hospital Peshawar, Peshawar, PAK

**Keywords:** developmental delay, hepatosplenomegaly, lysosomal storage disorder, inability to thrive, niemann-pick disease

## Abstract

Niemann-Pick disease is a rare lysosomal storage, autosomal recessive disorder that impairs the body's ability to metabolize fats, thus leading to accumulation within cells. It can affect various organs, most commonly the brain, liver, spleen, bone marrow and lungs. Hepatosplenomegaly, inability to thrive and varying neurological deficits are the defining features. The three main types of Niemann-Pick disease are: NPD-A (Niemann-Pick disease type A), NPD-B (Niemann-Pick disease type B) and NPD-C (Niemann-Pick disease type C). NPD-A and NPD-B are due to enzyme acid sphingomyelinase deficiency, caused by SMPD-1 (Sphingomyelin phosphodiesterase 1) gene mutation and NPD-C is due to NPC-1 and NPC-2 (Niemann-Pick C1 and C2 protein) gene mutation. This is the case report of an 11-month-old infant who presented to OPD (Outpatient Department) with failure to thrive, abdominal distension and developmental delay. On examination the infant was emaciated, pale, had hepatosplenomegaly and developmental delay. Bone marrow and liver biopsy showed characteristic lipid-laden foamy macrophages. Thus detailed history, examination and investigations confirmed NPD-A. NPD-A has a poor prognosis and is usually fatal by three years of age. The patient was provided supportive treatment like nutritional therapy and physiotherapy, and parents were counselled regarding the disease outcome. The patient is regularly followed up, and two episodes of chest infections were reported during an 8-month period of follow-up.

## Introduction

Niemann-Pick disease (NPD) is a lysosomal storage disorder that is autosomal recessive in inheritance and affects lipid metabolism. It affects the body’s ability to metabolize lipids particularly sphingomyelin, a type of fat found in cell membrane. Thus results in the buildup of sphingomyelin in different organs and tissues to a range of symptoms and complications. It is categorized into three main types: NPD-A, NPD-B and NPD-C, each with varying symptoms and rates of progression. NPD-A and NPD-B are due to enzyme acid sphingomyelinase deficiency caused by SMPD-1 gene (sphingomyelin phosphodiesterase 1) missense mutation, and NPD-C is due to NPC-1 and NPC-2 (Niemann-Pick C1 protein and Niemann-Pick C2 protein) gene mutation. NPD-A is a deadly illness of infancy, characterized by the inability to thrive, hepatosplenomegaly and a quickly progressing neurodegenerative course, that causes mortality by three years of age [1]. The incidence of NPD-A is 1 in 250000 in the general population but more prevalent in Ashkenazi Jews, which is predicted to be 1 in 40000 [2-5]. NPD-B is a non-neuropathic form, less severe and has a later onset, observed in both children and adults. It is characterized by the deposition of sphingomyelin in non-neuronal tissues including lungs, causing progressive lung disease. At diagnosis, NPD-B usually involves lungs with reticular or finely nodular infiltrations, visible on an X-ray chest. NPD-B has a better prognosis [2-4]. NPD-C is the most common neuropathic form, caused by NPC-1 and NPC-2 gene mutations, which provide instructions for the production of special proteins in lysosomes that are responsible for the transport of cholesterol. Onset is at two years of age but can occur in adults. It is characterized by ataxia, quadriparesis, seizures and moderate hepatosplenomegaly [2-4,6].

A patient with NPD-A is normal at birth, followed by hepatosplenomegaly and developmental delay, which become visible by six months of age. Overtime neuroregression begins with the loss of developmental milestones, such as the social smile, neck holding, and sitting, evident by the age of six months, followed by a decline in intellectual capabilities and the onset of spasticity [2-5].

## Case presentation

**Figure 1:**
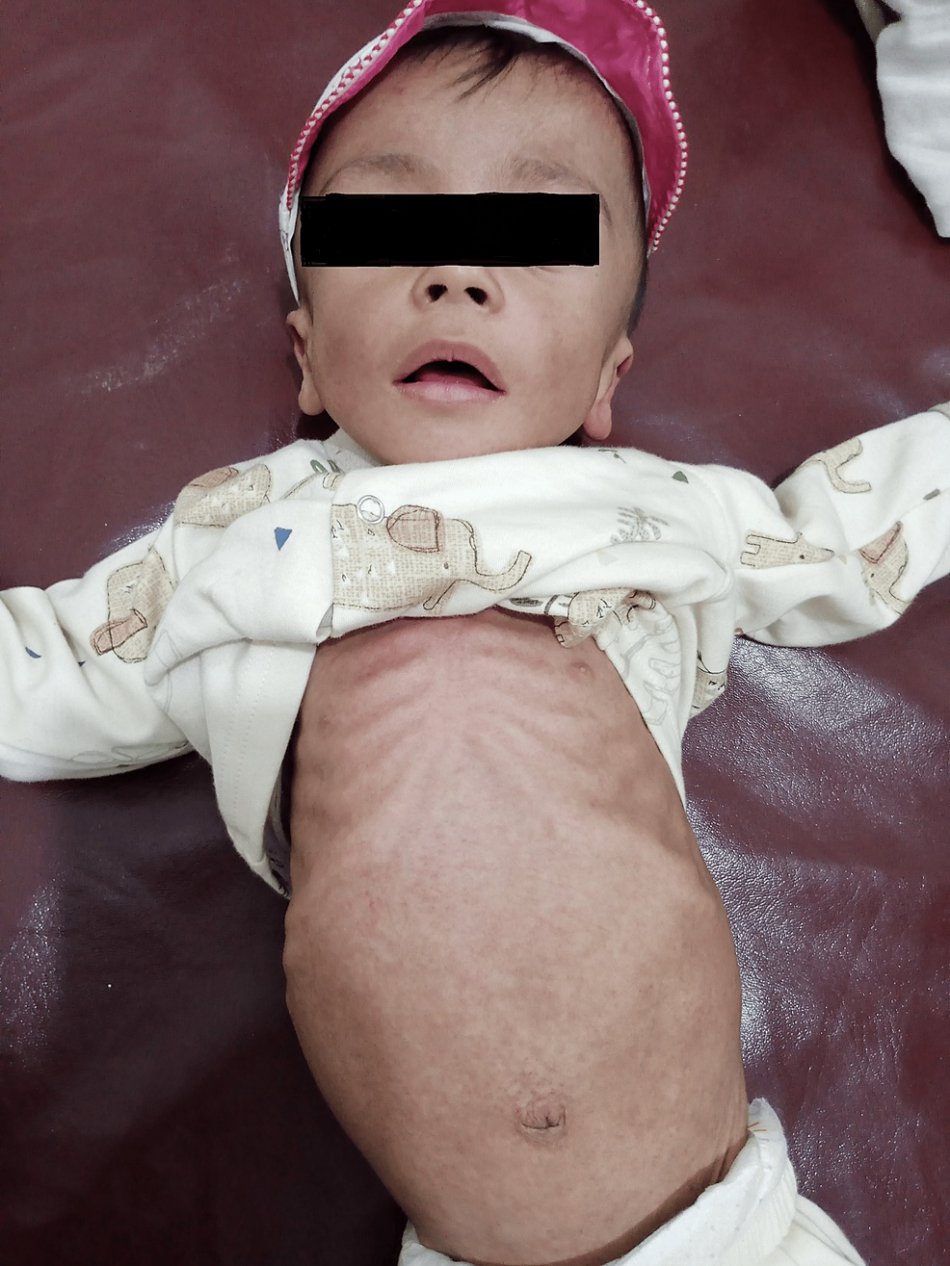
The photograph shows a severely wasted infant with coarse facial features and hepatosplenomegaly

**Figure 2:**
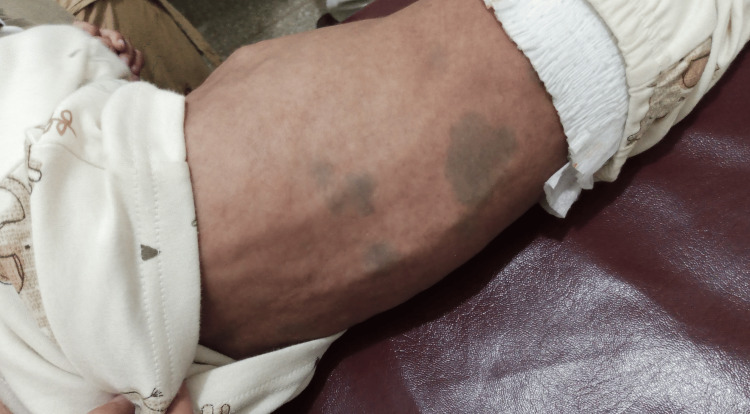
The photograph shows congenital dermal melanocytosis on the back

An 11-month-old male infant was brought to the OPD (Outpatient Department) of Lady Reading Hospital Peshawar, with the chief complaints of failure to thrive, gradual abdominal distension since the 5th month of age and developmental delay. The infant was 2nd issue of first-degree consanguineous marriage, born via spontaneous vaginal delivery at the 37th week of gestation, with a normal birth weight of 3.5 kg. Past medical history showed multiple hospital admissions for recurrent chest infections. Developmental history showed delayed milestones; social smile started at the 5th month of age, neck holding at the 6th month and sitting with support at the 7th month. After 7th month, the regression of milestones started with the loss of neck holding, sitting and social smile. He had positive transfusion history of packed red blood cells at the age of eight months, due to severe iron deficiency anemia. Vaccination history was up-to-date. On general physical examination, the patient was emaciated, pale and had coarse facies of nasal bridge depression and a prominent forehead (Figure 1). He had congenital dermal melanocytosis all over the body, more marked on the back (Figure 2). His height was 68 cm and weight was 5.3 kg, both below the 3rd percentile in the WHO (World Health Organization) growth chart. The patient was vitally stable with BP (Blood pressure) of 80/60 mmHg, PR (Pulse rate) of 68 beats per minute, RR (Respiratory rate) of 40 breaths per minute and temperature of 98 degrees Fahrenheit. The patient was unable to hold his neck, sit or roll over. Abdominal examination showed a distended abdomen on inspection. On palpation, the liver was enlarged 5 cm below the costal margin on the right side, with a total span of 10 cm. It was firm and non-tender. The spleen was enlarged 8 cm below the left costal margin. The neurological examination showed regression of developmental milestones. Eye movements were normal. The rest of the examination, including gastrointestinal, cardiovascular, and genitourinary assessments, was unremarkable.

**Figure 3:**
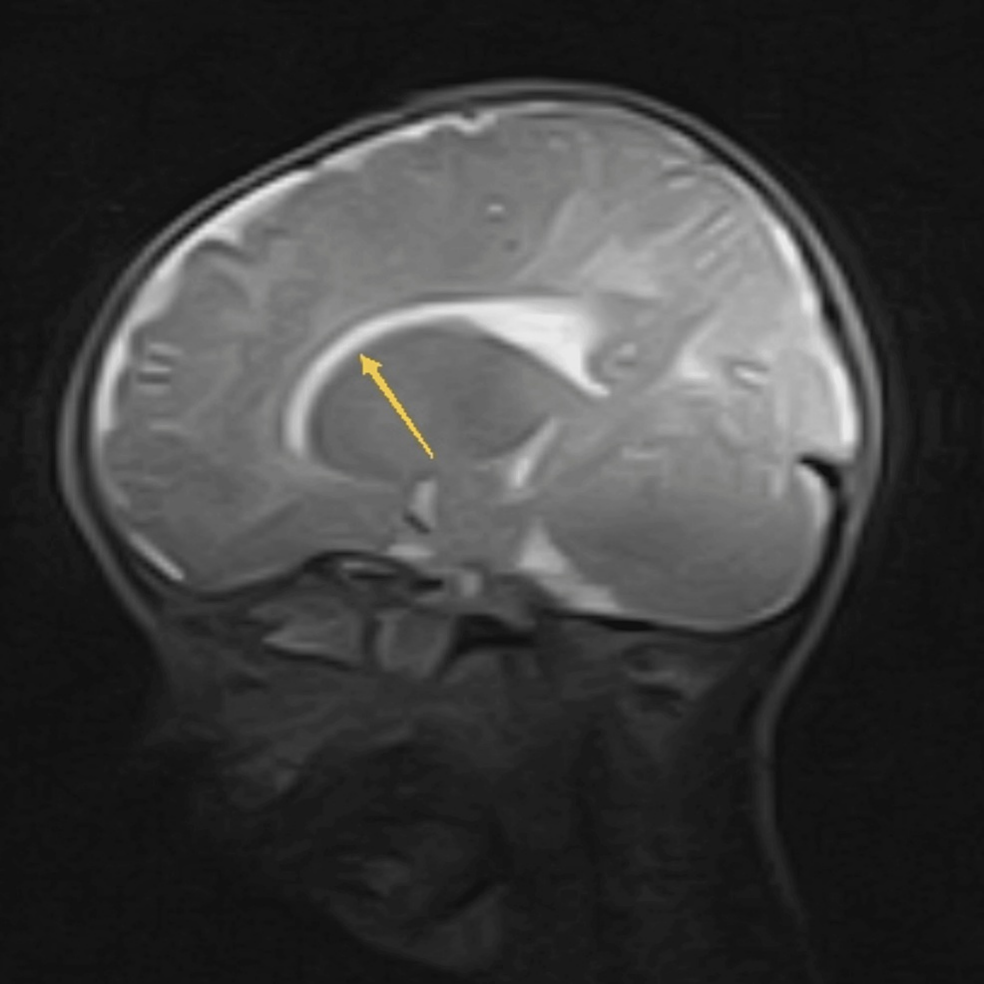
MRI brain T2W image shows thinning of corpus callosum (yellow arrow). MRI: Magnetic resonance imaging; T2W image: T2-weighted image

Complete blood count and peripheral smear showed hypochromic microcytic anemia with hemoglobin of 8.0 mg/dl, mean corpuscular volume 59.5 fl, mean corpuscular hemoglobin concentration 31.6 g/dl and packed cell volume 25.2%. Total leukocyte and platelet count were normal, 9000/cm^2^ and 2500000/cm^2^ respectively. Reticulocyte count was 2.5%. Liver function tests such as liver enzymes aspartate transaminase (AST), alanine transaminase (ALT), alkaline phosphatase (ALP), gamma-glutamyltransferase (GGT), serum albumin and total protein were normal. Serum electrolytes, random blood sugar, serum albumin, serum cholesterol, prothrombin time and thyroid function tests were within normal range. Chest X-ray was also normal. Ultrasound abdomen showed hepatosplenomegaly. On further workup, MRI (magnetic resonance imaging) of the brain revealed reduced hypointense signal in white matter bilaterally specially in subcortical regions, and also showed thinning of corpus callosum (Figure 3).

**Figure 4:**
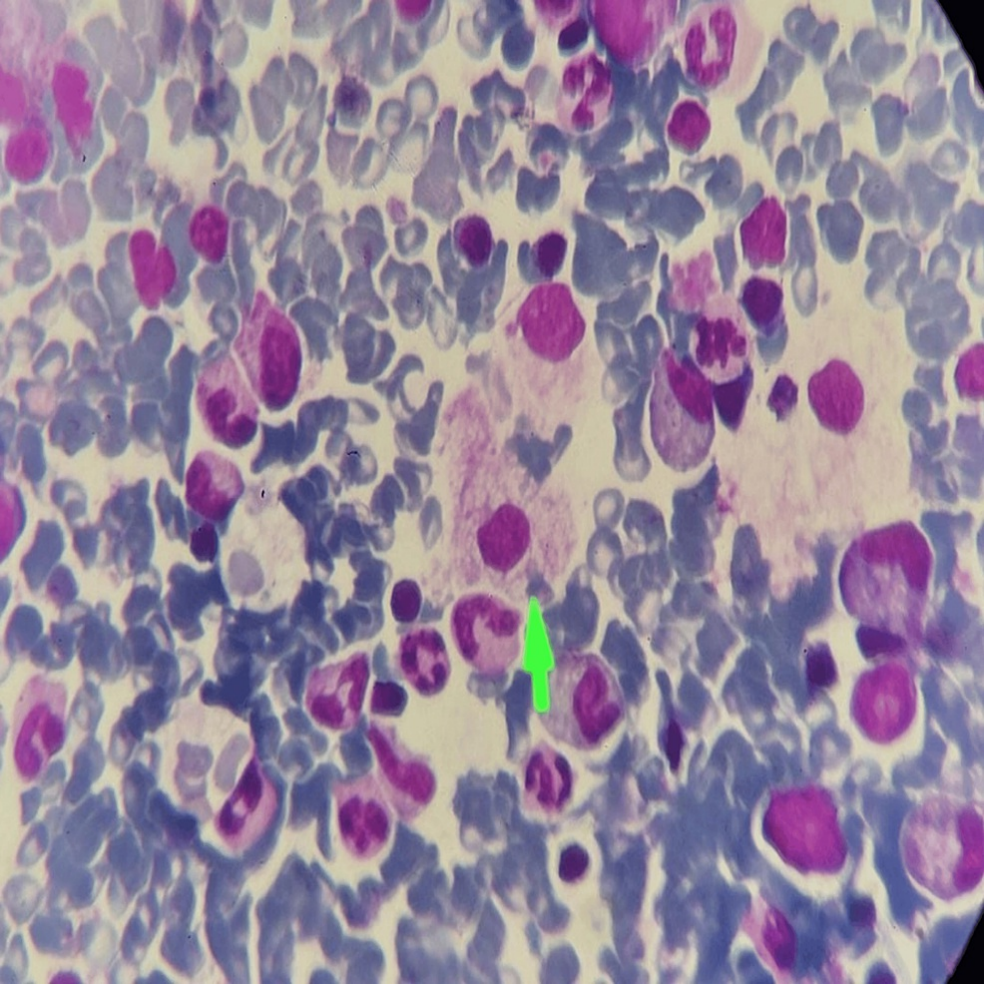
Bone marrow aspiration histopathological examination (100x) shows lipid-laden foamy histiocytes giving soap bubble appearance (green arrow), on Giemsa stain.

Bone marrow aspiration revealed lipid laden foamy histiocytes which are characteristic of Niemann-Pick disease (Figure 4). There was normoblastic erythroid hyperplasia and reduced iron (Table 1).

**Table 1:** Bone marrow aspiration report.

Parameter	Finding
Cellularity	Hypercellular
Erythropoiesis	Active and normoblastic
Megakaryocytes	Normal
Abnormal cells	Lipid-laden foamy histiocytes
Iron	Reduced

Fundoscopic examination didn’t show a macular cherry-red spot. Acid sphingomyelinase enzyme activity was advised but couldn’t be performed due to non-availability. Liver biopsy showed the same lipid-laden foamy histiocytes. Thus NPD-A diagnosis was made as a result of a thorough history, examination and investigations. This disease does not have a specific treatment; instead, supportive care is provided. The patient was symptomatically treated and parents were counseled regarding disease genetics, prognosis and its outcome. Our patient is now 19 months old and is regularly followed up, with two episodes of chest infections reported.

## Discussion

Niemann-Pick disease is a group of autosomal recessive disorders characterized by the buildup of lipids in the body including sphingomyelin and cholesterol. It is also called sphingomyelin-cholesterol lipidosis, caused by acid sphingomyelinase enzyme deficiency. Sphingomyelin and the precursor lipids accumulate in lysosomes, primarily in macrophages which deposit in the spleen, liver, brain and lungs thus resulting in hepatosplenomegaly, neurologic symptoms, lung diseases and cytopenias [4,7]. The three main types of Niemann-Pick disease are: NPD-A, NPD-B and NPD-C. NPD-A and NPD-B are due to missense mutations of the sphingomyelin phosphodiesterase 1 (SMPD 1) gene on chromosome 11p15 [4,7]. NPD-C is caused by mutation in NPC1 (95% of cases) and NPC2 (4% of cases) genes [6].

NPD-A is also known as the infantile neurovisceral form, it is due to very low activity of acid sphingomyelinase and is usually fatal by the age of three years. It manifests in the first few months of birth as growth retardation and hepatosplenomegaly. By the age of one year, regression of developmental milestones starts [2,4,7]. NPD-B has a later onset, observed in both children and adults and does not involve neuronal tissue. These patients develop hepatosplenomegaly and interstitial lung disease, which causes thrombocytopenia, recurrent chest infections and impaired bone growth [2,4,6]. NPD-C results from defective cholesterol transport, and typically affects children but can occur at any age. It causes quadriparesis, severe lung and liver illness, supranuclear gaze palsy, dysphagia, ataxia and dystonia [4,6,7]. NPD-A diagnosis is based on a detailed history, physical examination and laboratory investigations. Clinical features include failure to thrive, hepatosplenomegaly and developmental delay, which starts at three to six months of age. Neurological symptoms, like regression of developmental milestones and slowing of psychomotor abilities, become noticeable by the age of one year [8]. On fundoscopy, a macular cherry-red spot is seen in 50 percent of patients [4]. Laboratory investigations include peripheral smear, thyroid function tests and lipid profile. Bone marrow aspiration histopathology can help establish the diagnosis which shows lipid-laden foamy macrophages. For confirmation of diagnosis, acid sphingomyelinase activity is measured in leukocytes and genetic analysis is done for gene mutation [8,9].

This case report presents an 11-month-old male infant with classical features of Niemann-Pick disease type A, including failure to thrive, hepatosplenomegaly, and developmental delay. Failure to thrive, often observed in affected infants, results from the impaired metabolism and accumulation of sphingomyelin, which interferes with normal cellular functions. Hepatosplenomegaly is a common finding in Niemann-Pick disease due to the storage of lipids in the liver and spleen. Developmental delay is also a prominent feature of the disease, typically observed around six months of age [10]. In this case, the patient exhibited delayed milestones and subsequent regression, which is in line with the neurodegenerative course of NPD-A. The genetic basis of NPD-A involves mutations in the SMPD-1 gene, resulting in acid sphingomyelinase enzyme deficiency. This enzyme is responsible for breaking down sphingomyelin into ceramide and phosphocholine. The absence or reduced activity of acid sphingomyelinase leads to the buildup of sphingomyelin in various tissues and organs, causing the characteristic features of the disease. Although the acid sphingomyelinase enzyme activity assay was not carried out in our patient, the clinical presentation and the discovery of lipid-rich foamy histiocytes in the bone marrow and liver biopsies provide strong evidence for the diagnosis of NPD-A.

The prognosis of NPD-A is typically poor, with a life expectancy of around three years [9]. There is currently no specific treatment available for the disease, and management primarily focuses on supportive care to address the symptoms and complications associated with the disease [11]. Symptomatic treatment may include nutritional support, respiratory care, and physical therapy. The parents of the affected child should be provided with comprehensive counseling, explaining the genetic nature of the disease, its expected outcome, and the importance of ongoing supportive care. Early recognition and diagnosis can aid in providing appropriate supportive care and genetic counseling to the affected individual and their family. Since present therapies for NPD-A are only supportive in nature, more study is required to investigate new therapeutic possibilities. Additionally, raising awareness among healthcare professionals about the clinical features and diagnostic clues of Niemann-Pick disease can lead to earlier detection and intervention, potentially improving patient outcomes and quality of life.

## Conclusions

NPD-A is a fatal and rare, autosomal recessive disease which develops in the first few months of life and gets worse over time, causing mortality by three years of age. Despite the absence of a curative treatment for NPD-A, the management approach focuses on providing supportive care and optimizing the patient's quality of life. This involves a multidisciplinary team approach with the involvement of nutritionists, physiotherapists, and occupational therapists. Consanguinity is a substantial but changeable risk factor for NPD-A because it is an autosomal recessive disease. Further research is needed to advance our understanding of NPD-A and develop potential therapeutic interventions to improve outcomes for patients with this devastating condition. Gene treatments and enzyme replacement therapies are being tested, and they may eventually replace other forms of treatment as the mainstay. This case report highlights the clinical features, diagnostic workup, and management approach for NPD-A, contributing to the understanding and awareness of this devastating condition among the healthcare professionals.

## References

[1] Aghamahdi F, Nirouei M, Savad S (2022). Niemann-Pick type A disease with new mutation: a case report. J Med Case Rep.

[2] Tangde A, Pore S, Kulkarni A, Joshi A, Bindu R (2017). Niemann-Pick disease type A-a case report. Int J Res Med Sci [Internet].

[3] (2024). National Institute of Neurological Disorders and Stroke (NINDS). Niemann-Pick disease. https://www.ninds.nih.gov/Disorders/All-Disorders/Niemann-Pick-Disease-Information-Page.

[4] Jeon EY, Choi KA, Koo CH (1998). A case of type A Niemann-Pick disease. J Korean Pediatr Soc.

[5] Kundu GK, Anwar SS, Liza NAS (2023). Niemann Pick disease: a rare lysosomal storage disease. Bangabandhu Sheikh Mujib Med Univ J.

[6] Vanier MT (2010). Niemann-Pick disease type C. Orphanet J Rare Dis.

[7] Vélez Pinos PJ, Saavedra Palacios MS, Colina Arteaga PA, Arevalo Cordova TD (2023). Niemann-Pick disease: a case report and literature review. Cureus.

[8] Qureshi K, Abdulmajeed ZG, Saleem S, Tariq J, Iqbal M (2022). Niemann-Pick disease type A: a rare disease with a fatal outcome. Cureus.

[9] Rajkumar V, Dumpa V (2024). Lysosomal storage disease. In: StatPearls [Internet].

[10] Wasserstein M, Dionisi-Vici C, Giugliani R (2019). Recommendations for clinical monitoring of patients with acid sphingomyelinase deficiency (ASMD). Mol Genet Metab.

[11] Shubhankar M, Sunil KA, Bikash RP, Shantanu KM (2014). Niemann Pick disease type A in an Infant: a case report. Sch Acad J Biosci.

